# Dosimetric and radiobiological comparison of treatment plan between CyberKnife and EDGE in stereotactic body radiotherapy for pancreatic cancer

**DOI:** 10.1038/s41598-021-83648-5

**Published:** 2021-02-18

**Authors:** Zhi-tao Dai, Li Ma, Ting-ting Cao, Lian Zhu, Man Zhao, Ning Li

**Affiliations:** 1 National Cancer Center/National Clinical Research Center for Cancer/Cancer Hospital and Shenzhen Hospital, Chinese Academy of Medical Sciences and Peking Union Medical College, Beijing, China; 2 Tongji Hospital, Tongji Medical College, Huazhong University of Science and Technology, Wuhan, 430030 China; 3 Department of Radiation Oncology, Shanghai East Hospital, Tongji University, Shanghai, 200120 China

**Keywords:** Cancer therapy, Radiotherapy, Cancer

## Abstract

To perform a comparison of the different stereotactic body radiotherapy (SBRT) plans between the Varian EDGE and CyberKnife (CK) systems for locally advanced unresectable pancreatic cancer. Fifteen patients with pancreatic cancer were selected in this study. The median planning target volume (PTV) was 28.688 cm^3^ (5.736–49.246 cm^3^). The SBRT plans for the EDGE and CK were generated in the Eclipse and Multiplan systems respectively with the same contouring and dose constrains for PTV and organs at risk (OARs). Dose distributions in PTV were evaluated in terms of coverage, conformity index (CI), new conformity index (nCI), homogeneity index (HI), and gradient index (GI). OARs, including spinal cord, bowel, stomach, duodenum and kidneys were statistically evaluated by different dose-volume metrics and equivalent uniform dose (EUD). The volume covered by the different isodose lines (ISDL) ranging from 10 to 100% for normal tissue were also analyzed. All SBRT plans for EDGE and CK met the dose constraints for PTV and OARs. For the PTV, the dosimetric metrics in EDGE plans were lower than that in CK, except that D_99_ and GI were slightly higher. The EDGE plans with lower CI, nCI and HI were superior to generate more conformal and homogeneous dose distribution for PTV. For the normal tissue, the CK plans were better at OARs sparing. The radiobiological indices EUD of spinal cord, duodenum, stomach, and kidneys were lower for CK plans, except that liver were higher. The volumes of normal tissue covered by medium ISDLs (with range of 20–70%) were lower for CK plans while that covered by high and low ISDLs were lower for EDGE plans. This study indicated that both EDGE and CK generated equivalent plan quality, and both systems can be considered as beneficial techniques for SBRT of pancreatic cancer. EDGE plans offered more conformal and homogeneous dose distribution for PTV, while the CK plans could minimize the exposure of OARs.

## Introduction

Pancreatic cancer is the fourth leading cause of cancer-related mortality worldwide with a 5-year survival rate approximately 20%^1,2^. For localized disease, surgery with complete resection represents the only potential treatment option associated with any substantive chance of cure^3,4^. However, due to non-specific early symptoms and aggressive behavior of pancreatic cancer, most patients were diagnosed at relatively late stages^5^. Most studies have demonstrated that chemotherapy combined with radiation therapy is more effective than single-modality therapy, despite continuous controversies about the role of radiation therapy exist due to conflicting clinical outcomes^6–9^. Surrounded by many important and radiosensitive gastrointestinal organs,

such as duodenum and stomach, the conventional radiotherapy for pancreatic cancer seems not to effectively spare these organs while delivering high dose to target^7^.

Compared with three dimensional conformal radiotherapy (3DCRT), intensity modulated radiotherapy (IMRT) can reduce the dose of normal organs surrounding tumor, and also minimize the toxicity of gastrointestinal organs^10^. Goto and Colbert^11,12^ had performed IMRT and 3DCRT for local pancreatic cancer, and compared dosimetry and clinical outcomes. They verified.

that IMRT offered better dose constrains for target and organs at risk (OAR) compared to 3DCRT. Brown and Coworkers^13^ also demonstrated that with the technology of IMRT, the prescription dose could be increased to 64.8 Gy, while maintained dose limits of OARs including spinal cord, liver, kidneys, and small bowel.

Although the IMRT provided a probability of better tumor control for locally pancreatic cancer, the organ motion and patient set-up errors during the treatment may affect the radiation dose of organs due to the complex site of OAR surrounding tumor^14^. As a new technique born of the synthesis of all of the above-mentioned advances, stereotactic body radiotherapy(SBRT) is becoming more widespread, and it is probably known as a promising method of radiotherapy for pancreatic cancer with greater normal tissue sparing^15–18^. With higher dose per fraction, the dose gradient of SBRT plans is steeper than other conventional radiation, and has better sparing of normal structures^19^. Lin et al. demonstrated the SBRT have the advantage of improving the local control for pancreatic cancer compared to the IMRT^20^. Kumar et al.^21^ similarly made a dosimetric analysis of the SBRT plans with duodenal sparing using volumetric-modulated arc therapy (VMAT) and IMRT in locally advanced pancreatic cancer. SBRT plans usually applied non-coplanar field arrangement, especially for the CyberKnife (Accuray, Inc, Sunnyvale) system^22^. With 6D robotic arm and accurate tracking techniques, the CyberKnife, a 6 MV linear accelerator, has high precision for dose delivery with a large degree of freedom, and the capability of real-time tumor positioning and correction.

As a culmination in the field of radiosurgery, EDGE (Varian Medical Systems, Palo Alto, CA) has advantages of safety, noninvasive, comfortable radiosurgery in the treatment of new experience. The general application of EDGE is the intracranial SRS technique, which can eliminate small lesions of intracranial accurately. Another application is the SBRT technique of real-time tracking and dynamic irradiation technology, focusing on body dynamic target area constantly^23^. This machine is equipped with flattening filter (FF) and flattening filter free (FFF) beams, and the high resolution multi-leaf collimators (MLC) of 120 leaves with 2.5 mm widths at the iso-center^24–26^. Thus it could deliver higher dose rates more effectively and accurately while improving the conformity of dose distribution to the target simultaneously^25^.

Currently, there is no study directly comparing dose distributions of SBRT plans between the CyberKnife(CK) and EDGE systems. In our study, two series of SBRT plans were generated using CK and EDGE platforms, respectively. We evaluated the different dosimetric metrics for target and normal tissue, as well as analyzing the radiobiological indices to reflect the response of radiation therapy.

## Materials and methods

### Patients data

This study performed a retrospective analysis of patients with pancreatic cancer who had undergone SBRT. 15 patients with locally advanced unresectable pancreatic cancer were included in this study. The inclusion criteria were as follows: (1) diagnose confirmed by pathological examinations; (2) locally advanced pancreatic cancer; (3) unresectable cancer intolerant of surgical resections; (3) age ranging from 18 to 75 years; (4) receiving the prescription dose of 6.5, 7.0 and 7.5 Gy × F; (5) ≥ 95% of PTV covered by prescription dose. The following exclusion criteria were used: (1) patients with a history of radiotherapy prior to the SBRT; metastatic pancreatic cancer.

Fiducial implantation will be done under endoscopic ultrasonography guidance. The number of implanted fiducials is 3 (at least 1) which is preferable to be close to, but not in the tumor. A time-period of 4–7 days between implantation and treatment planning CT-scan is applied.CT simulation was performed with head first supine position on a Brilliance Big Bore 16-slice CT scanner (Philips, Amsterdam, the Netherlands) with a slice thickness of 1.5 mm. Gross target volume (GTV) and critical structures including spinal cord, bowel, stomach, duodenum and kidneys were contoured jointly by oncologist and radiologist based on the fusion of CT and magnetic resonance (MR) images on the MultiPlan system (Accuray Inc., Sunnyvale CA; version 4.02). Planning target volume (PTV) were defined by expanding the GTV with 2 or 3 mm margin in all directions. The median of GTV was 18.79 cm^3^ (ranged from 2.67 to 34.73), and that of the PTV was 28.688 cm^3^ (ranged from 5.736 to 49.246). The critical normal tissue include spinal cord, bowel, stomach, duodenum, left kidney, right kidney and spleen were outlined according the Radiation Therapy Oncology Group (RTOG) for pancreatic cancer^27^. All methods were carried out in accordance with relevant guidelines and regulations. Informed Consent was obtained from the patients for study participation. Consents for publication of data have been obtained from all patients. All the patients included in this study are above 18 years old.

### SBRT planning

According to the different prescribed dose, fifteen patients were divided into three groups, and each group consisted of five patients. The prescription dose for the three groups were of 37.5 Gy/5F, 35.0 Gy/5F, 32.5 Gy/5F, respectively. The dose was prescribed to ~ 70% isodose line relative to maximum dose of PTV. After importing all image data of 15 patients into two systems, CyberKnife (CK) and EDGE, different SBRT treatment plans were designed by the same medical physicists The dose constrains of targets and normal tissue were set to meet the criteria of the RTOG 0848 and the report of AAPM Task Group No. 101 (AAPM TG-101)^28–30^, as shown in Table 1.

**Table 1:** Dose constrains of target and normal tissue for SBRT plans.

Structure	Metrics	Objective
PTV	V_100_ (%)	≥ 95%
	PIDL	~ 70%
Spinal Cord	D_max_ (Gy)	< 27 Gy
	D_0.25cc_ (Gy)	< 22.5 Gy
	D_1.2cc_ (Gy)	13.5 Gy
Duodenum	D_max_ (Gy)	32 Gy
	D_5cc_ (Gy)	< 18 Gy
	D_10cc_ (Gy)	< 12 Gy
Bowel	D_max_ (Gy)	< 35 Gy
	D_5cc_ (Gy)	< 19.5 Gy
Stomach	D_max_ (Gy)	< 32 Gy
	D_10cc_ (Gy)	< 18 Gy
Liver	V_<17.5Gy_ (cc)	> 700 cc
Left kidney	D_mean_ (Gy)	< 12 Gy
	V_>23Gy_ (%)	< 66.7%
Right kidney	D_mean_ (Gy)	< 12 Gy
	V_>23Gy_ (%)	< 66.7%
Spleen	No constraint	

The CK plans were designed for G4 system with Multiplan TPS (version 4.0.2). The 6 MV FFF photon beam was applied and dose rate was set to 800 MU/min with one or two cones with size of 10–30 mm. Beside the dosimetric constraints listed in Table 1, five ‘shells’ expanded isotropically from PTV were used to make steep dose fall-off gradient. At the end of the optimization, beams and time reduction were used to make the plan clinically practical. All CK plans were optimized using the sequential process with the ray tracing algorithm. Method of 1 fiducial plus X-sight spine and Synchrony Tracking technique were applied.

The plans for EDGE system were generated with the Varian Eclipse system (Varian Medical Systems, Palo Alto, CA; version 13.5). A VMAT plan for each case was generated using two 360°arcs with the same iso-centre at the geometric centre of PTV. The 10 MV FFF photon beam was chosen with a high dose rate of 2400 MU/min. All VMAT plans were optimized using the progressive resolution optimizer (PRO) and analytical anisotropic algorithm (AAA) with a grid size of 1.5 mm were applied in dose calculation. In order to make the plan comparisons valid, both CK and EDGE plans were nornalized to ensure ≥ 95% of PTV covered by prescription dose.

### Evaluation metrics of PTV

As were listed in Tables 1 and 2, the coverage and mean dose (D_mean_) of PTV, as well as doses covered 99%, 95%, 5% and 1% of PTV (D_99_, D_95_, D_5_, D_1_) of PTV were categorized for plan evaluation. Meanwhile, the conformity index (*CI*), new conformity index (*nCI*), homogeneity index (HI), and gradient index (GI) were also used to quatify the plan quality. *CI* and *nCI* quatifying the target coverage and healthy tissue sparing were defined as follow^31^:

1 $$ CI = \frac{{V^{{R_{x} }} }}{{V_{PTV}^{{R_{x} }} }} $$

2 $$ nCI = \left( {\frac{{V^{{R_{x} }} }}{{V_{PTV}^{{R_{x} }} }}} \right)/\left( {\frac{{V_{PTV}^{{R_{x} }} }}{{V_{PTV} }}} \right) $$

where the $V^{{R_{x} }}$ is the volume covered by prescription isodose line (PIDL), $V_{PTV}$ is the target volume, and the $V_{PTV}^{{R_{x} }}$ is the volume of target covered by PIDL. Smaller CI and nCI imply a more conformal plan and the ideal values for both indices are 1.0.

**Table 2:** The dosimetric indexes comparison of PTV between Cyberknife and EDGE plans.

Metrics	CK + SD	Edge + SD	*p*
V_100_ (%)	96.8 ± 10.84	95.04 ± 0.03	0.000
D_mean_ (%)	123.91 ± 1.97	112.32 ± 3.39	0.000
D_99_ (%)	93.28 ± 2.53	97.13 ± 0.64	0.000
D_95_ (%)	102.92 ± 1.40	100.02 ± 0.01	0.000
D_5_ (%)	137.83 ± 2.30	125.40 ± 7.13	0.000
D_1_ (%)	139.42 ± 2.04	129.27 ± 7.42	0.000
CI	1.184 ± 0.076	0.986 ± 0.019	0.000
nCI	1.222 ± 0.072	1.037 ± 0.020	0.000
HI	0.416 ± 0.033	0.296 ± 0.077	0.000
GI	3.070 ± 0.222	4.145 ± 0.312	0.000

The homogeneity index which mainly used to evaluate the degree of the dose uniformity inside the target volume^32^ was defined as Eq. (3):

3 $$ HI = \frac{{D_{2} - D_{98} }}{{D_{P} }} $$

where the $D_{x}$ is the dose that covers *x* percent volume of PTV, and the $D_{p}$ is the prescription dose of target. Usually, *HI* > 0, and *HI* = 0 means each voxel of target volume receives the same dose.

At the same time, in order to assess the degree of dose fall-off outside the target volume, the gradient index has been applied, which is calculated according to the following equation^33^:

4 $$ GI = \frac{{V_{50\% } }}{{V_{100\% } }} $$

where the V_x%_ is the absolute volumes covered by x% of PIDL. For SBRT plan, smaller value of *GI* means steeper dose fall-off and better normal tissue sparing.

### Evaluation metrics of OARs

The maximum dose (D_max_) and mean dose (D_mean_) of all the contoured OARs were accessed. Moreover, organ specialized DVH metrics, for instance D_0.25cc_ and D_1.2cc_ of spinal cord, were also evaluated according to AAPM TG-101. The details of OAR evaluation metrics were listed in Table 2. At the saome time, equivalent uniform dose (EUD) was applied to convert the heterogeneous dose distributions into homogeneous dose. Based on the phenomenological model introduced by Niemierko, the EUD is defined as follows^34^:

5 $$ EUD = \left( {\sum\limits_{i = 1} {v_{i} EQD_{i}^{a} } } \right)^{1/a} $$

where *v*_*i*_ is the percentage of voxels receiving dose *D*_*i*_. The *v*_*i*_ and *D*_*i*_ values are acquired from the DVHs and the sum of *v*_*i*_ over all voxels equals 1. And parameter ‘*a*’ denotes the seriality property for different organs, and is usually set to a positive value for OARs. In reference^35^ a parameter *n* = *1/a* was used. The EQD is calculated as follows, which is defined as biologically equivalent dose of 2 Gy per fraction:

6 $$ EQD = D \times \frac{{\left( {\frac{\alpha }{\beta } + \frac{D}{n}} \right)}}{{\left( {\frac{\alpha }{\beta } + 2} \right)}} $$

where *n* denotes the number of fractions, and *α/β* is a parameter from the issue-specific Linear Quadratic (LQ) model of the certain organ, determining the fractionation sensitivity. The values of parameters a and a = b were listed in Table 4 according to reference^35^.

### Volumes covered by different ISDL

To analyze the details of dose distribution outside PTV, the absolute volumes of normal tissue that covered by *x* percent of prescription isodose lines (*V*_*x*_) ranging from 100 to 10% with intervals of 10% were compared between CK and EDGE plans. Ratios between volumes of normal tissue (*V*_*x*_) and PTV (*V*_*PTV*_) were also calculated to minimize the effect resulted from different PTV volumes. Meanwhile, effective distance $\Delta R_{Eff}$ was applied to quantify the dose fall-off details of different ISDL, which is defined as follow:

7 $$ \Delta R_{Eff} = R_{iso}^{x} - R_{PTV} $$

where $R_{iso}^{x}$ and $R_{PTV}$ were the equivalent radius of spheres with volumes of *V*_*x*_ and *V*_*PTV*_, which were calculated based on sphere volume formula *V* = *4πR*^*3*^*/3.*

### Statistical analysis

For those 15 patients with two different SBRT plans in EDGE and CK systems, a paired t-test statistical analysis were performed using the IBM SPSS statistical software version 21 (SPSS Inc., Armonk, NY) to determine the difference, and if *P* value < 0.05, it was consider to have the statistical significance. All datas were listed in terms of mean value ± standard deviation (SD).

### Ethics approval and consent to participate

The study was approved by the institutional review board of National Cancer Center/National Clinical Research Center for Cancer/Cancer Hospital & Shenzhen Hospital. We confirm that all methods were carried out in accordance with relevant guidelines and regulations.

### Consent for publication

The consents for publication of data have been obtained from patients.

## Results

In total, a retrospective analysis of 15 patients with pancreas cancer was performed. The treatment plans of SBRT were designed in CK and EDGE systems, respectively. Plans generated in both platforms could meet the clinical criteria of PTV coverage and OAR sparing. The median volume of tumor was 28.688cm^3^ (5.736–49.246 cm^3^). All of CK and EDGE plans were normalized to ensure at least 95% of PTV covered by prescription dose.

### Evaluation of PTV

**Figure 1:**
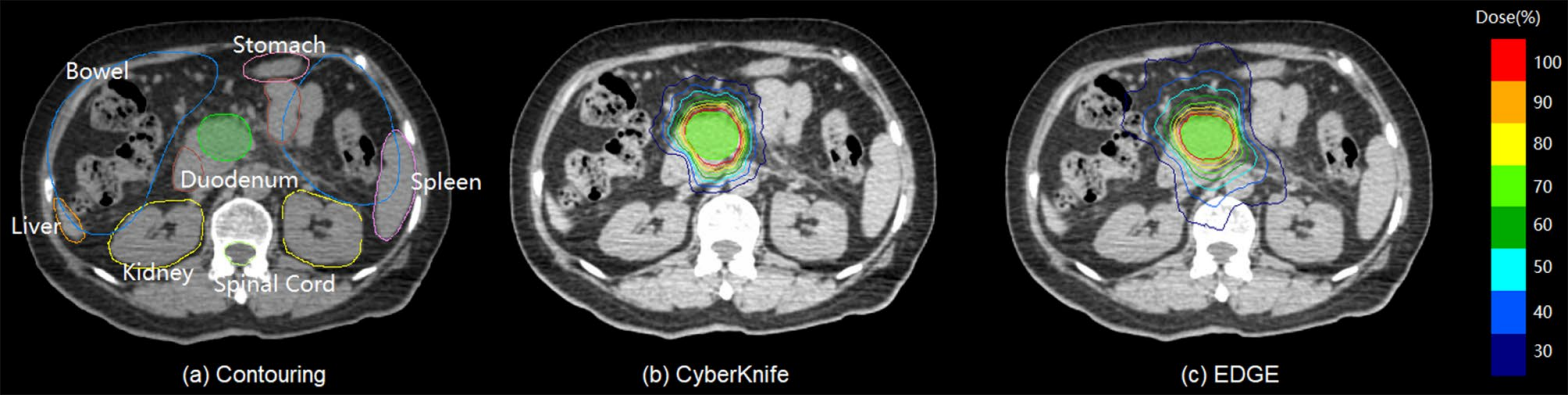
Contouring and comparison of planar dose distribution for one selected case. (**a**) Is contouring of target and OARs, and (**b**),(**c**) are planar dose distribution of CyberKnife and EDGE plans, respectively.

The comparison of isodose lines from 30 to 100% of the prescription dose for a selected case was illustrated in Fig. 1. It reveals that both plans have excellent conformity and adequate coverage for PTV. Besides, we can find that the 100% PIDL (with red color) of EDGE plan is closer to PTV boundary than that of CK plan.

**Figure 2:**
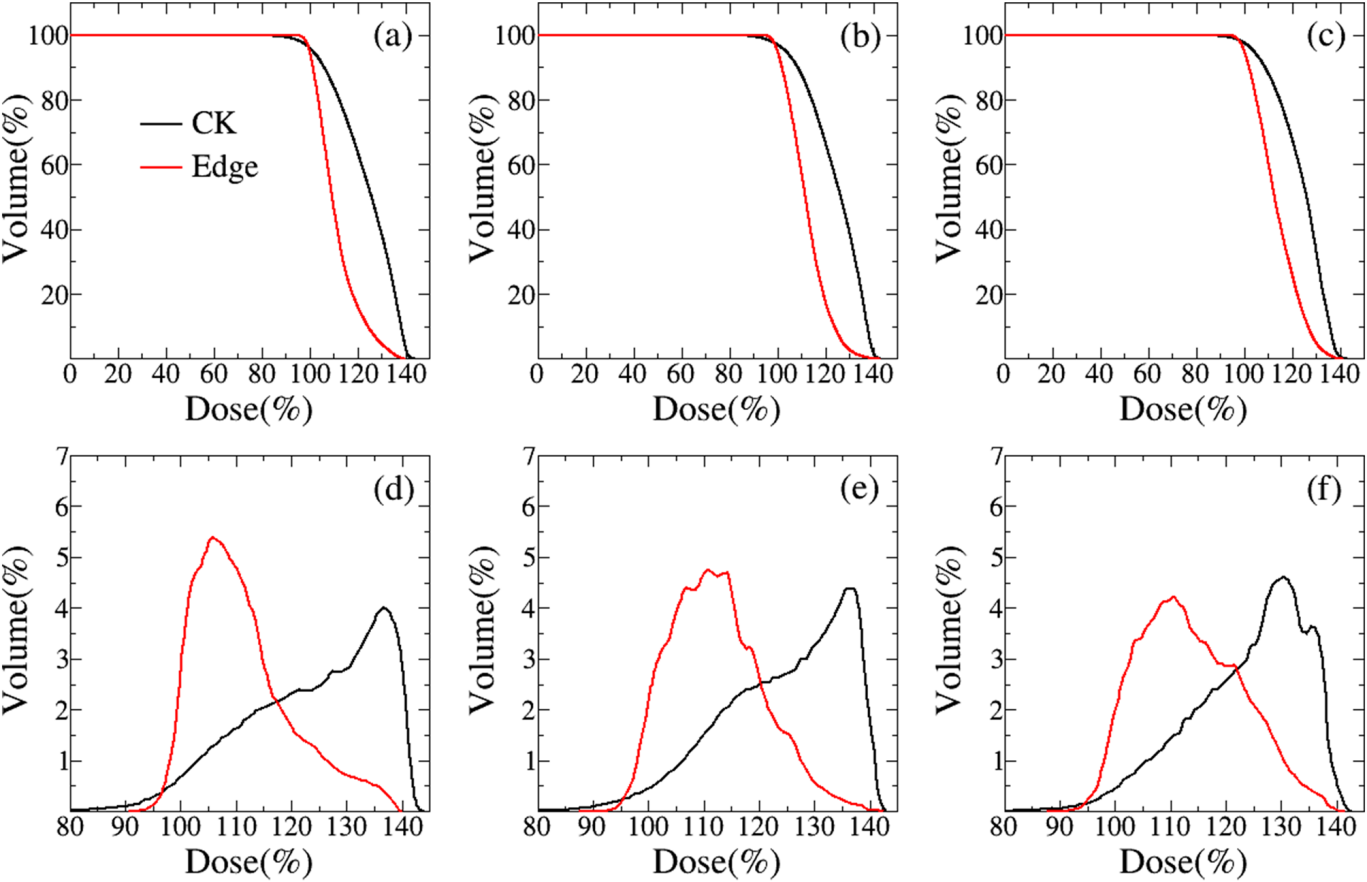
The average DVHs curves of PTV for plans with prescription dose of 37.5 Gy/5F (left column), 35.0 Gy/5F (middle column) and 32.5 Gy/5F (right column). The upper and lower rows represent the integral and differential DVHs, respectively. The black line is for CK, and the red line is for EDGE.

The average DVHs of PTV for CK and EDGD plans are shown in Fig. 2. From integral DVHs displayed in the upper row of Fig. 2, we have found that PTV coverage of EDGE plans are a little higher than that of CK in all the three groups. Further, we have investigated the details of PTV DVHs in the way of differential as were displayed in the lower row of Fig. 2, from which we may conclude that the voxel dose of EDGE plans are more closed to prescription dose than the one of CK. It is also indicated that both of the cold and hot point volumes of CK plans larger than those of EDGE plans. This means that EDGE plans are more conformal and homogeneous.

**Figure 3:**
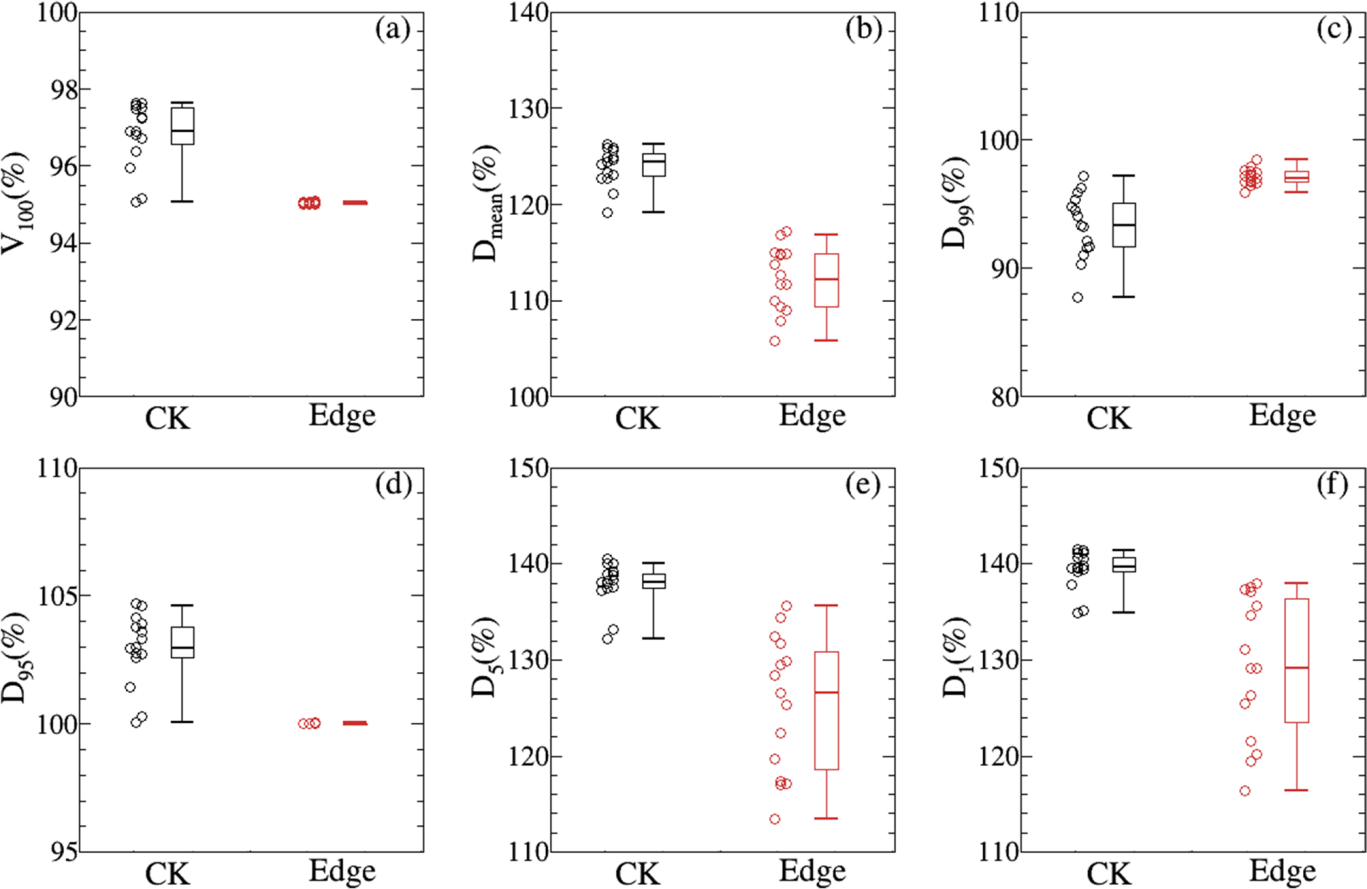
Comparison of different dosimetric metrics of PTV. (**a**)–(**f**) Is for V_100_, D_mean_, D_99_, D_95_, D_5_ and D_1_, respectively. The black line is for CK, and the red line is for EDGE.

**Figure 4:**
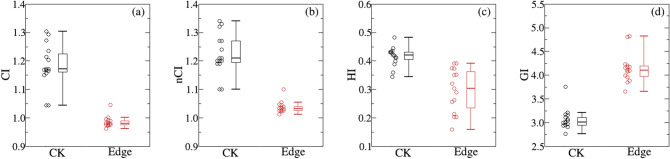
Comparison different evaluationindexes of PTV for EDGE and CK plans. (**a**) Conformal index (CI), (**b**) new conformal index (nCI), (**c**) homogeneity index (HI), (**d**) gradient index (GI). The black color is for CK, and the red color is for EDGE.

The dosimetric metrics of PTV including V_100_, D_mean_, D_99_, D_95_, D_5_ and D_1_ are displayed in Fig. 3 and Table 2. To ensure cases with different prescription doses are comparible, all of the dose-volume metrics are expressed with percentage values. It is indicated that PTV coverage (V_100_) is slightly higher for CK, which may results from different normalization methods. Dosimetric metrics including D_mean_, D_99_, D_95_, D_5_ and D_1_ are smaller for EDGE except that D_99_ is a little higher compared with those of CK plans. This is consistent with Fig. 2. Other evaluation indexes such as CI, nCI, HI and GI are displayed in Fig. 4a–d, and the statistical data is detailed in Table 2. The CI and nCI of PTV for EDGE plans are 0.986 ± 0.019,1.037 ± 0.020, respectively, which are smaller than those of CK plans with 1.184 ± 0.076 and 1.222 ± 0.072(as shown in Table 2). And HI of both plans are also compared, from which the values of 0.296 ± 0.077 and 0.416 ± 0.033 are obtained for EDGE and CK, respectively. It can be concluded that EDGE plans are superior in terms of conformity and homogeneity. However, GI for CK plans are more lower than EDGE, which implies the steeper dose fall-off gradient.

### Evaluation of OARs

**Figure 5:**
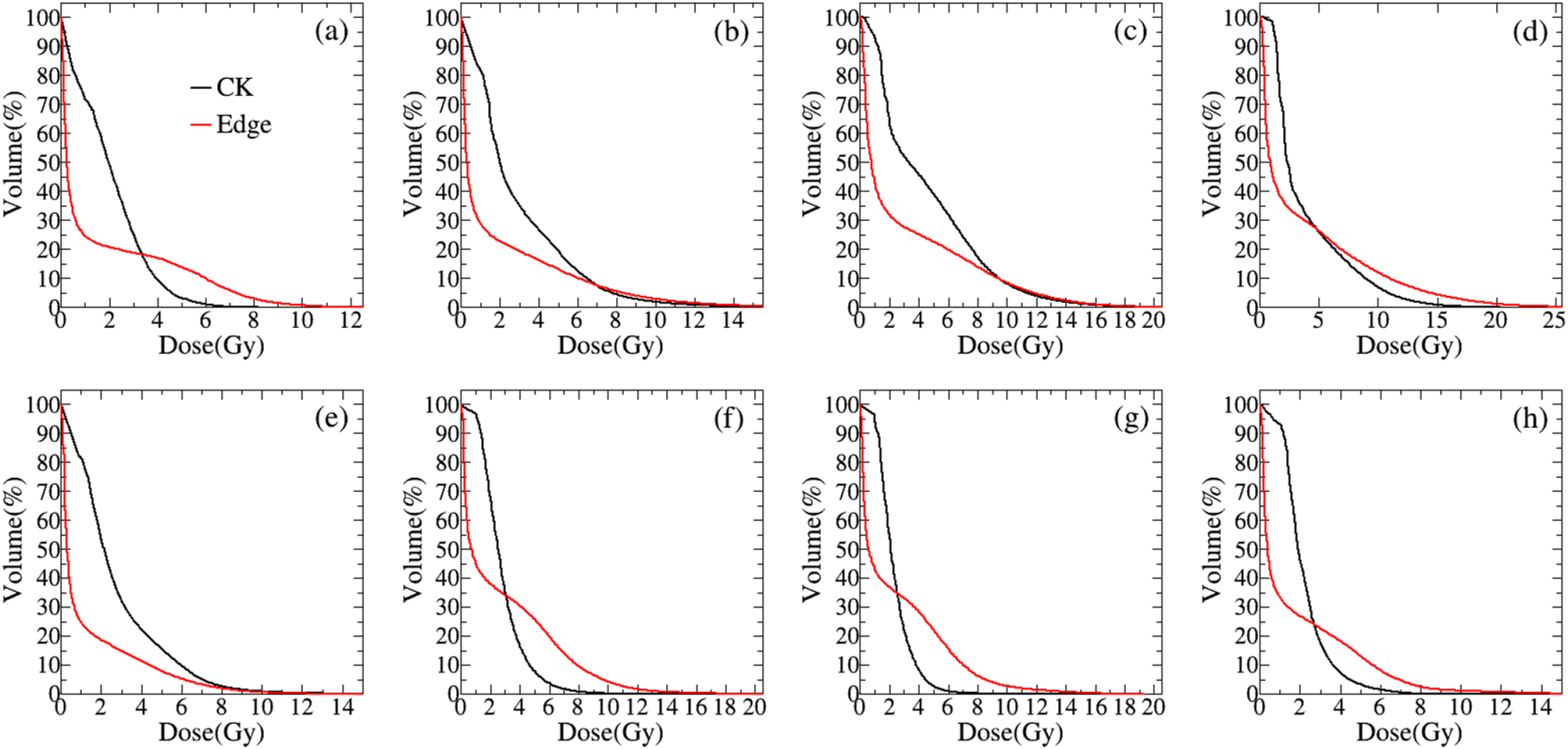
The average DVH curves of normal tissue adjacent to tumor: (**a**) spinal cord, (**b**) duodenum, (**c**) bowel, (**d**) stomach, (**e**) liver, (**f**) left kidney, (**g**) right kidney, and (**h**) spleen. The black line is for CK, and the red line is for EDGE.

The average DVHs of organs at risk including spinal cord, bowel, stomach, duodenum, Liner, left kidney, right kidney and spleen are displayed in Fig. 5a–h. And Table 3 shows the results of dose-volume parameters of normal tissue. All criteria of the dose constrain for normal tissue were achieved in both systems. Compared with CK plans, the dosimetric metrics of spinal cord including D_max_, D_0.25cc_, D_1.2cc_ were slightly higher for EDGE plans with significant statistical differences, which indicates the decreased sparing of spinal cord with EDGE. From Table 3, the D_5cc_ of bowel and the mean dose of bowel, stomach, liver, and kidneys are slightly lower for EDGE plans with statistic difference (*p* < 0.001), but other dose-volume metrics shows no difference.

**Table 3:** The dosimetric metrics comparison of OARs between Cyberknife and EDGE plans.

Structure	Metrics	CK + SD	Edge + SD	*p*
Spinal Cord	D_max_	5.69 ± 1.62	9.22 ± 2.04	0.000
	D_mean_	1.97 ± 0.53	1.45 ± 0.46	0.000
	D_0.25cc_	5.12 ± 1.49	8.39 ± 1.79	0.000
	D_1.2cc_	4.62 ± 1.30	7.60 ± 1.62	0.000
Duodenum	D_max_	16.22 ± 6.36	19.89 ± 5.95	0.140
	D_mean_	3.61 ± 1.43	3.49 ± 1.64	0.847
	D_5cc_	8.34 ± 3.71	11.15 + 5.59	0.143
	D_10cc_	6.66 ± 3.18	8.58 ± 5.13	0.271
Bowel	D_max_	19.98 ± 5.25	20.56 ± 2.80	0.516
	D_mean_	2.81 ± 0.75	1.63 ± 0.59	0.000
	D_5cc_	13.39 ± 0.91	15.26 ± 2.77	0.001
Stomach	D_max_	20.68 ± 4.87	21.57 ± 6.52	0.422
	D_mean_	4.49 ± 1.73	2.82 ± 1.62	0.000
	D_10cc_	11.82 ± 2.96	11.07 ± 4.32	0.347
Liver	D_mean_	2.67 ± 1.23	1.23 ± 0.20	0.000
	V_<17.5Gy_(cc)	1299.07 ± 251.72	1299.07 ± 252.67	0.306
Left kidney	D_mean_	2.21 ± 0.76	0.39 ± 0.20	0.000
	V_>23Gy_ (%)	2.80 ± 0.89	2.75 ± 1.09	0.798
Right kidney	D_mean_	1.79 ± 0.49	0.48 ± 0.67	0.000
	V_>23Gy_ (%)	2.30 ± 0.62	2.47 ± 1.15	0.337
Spleen	D_max_	7.56 ± 2.20	8.23 ± 3.60	0.450
	D_mean_	2.16 ± 0.77	1.70 ± 1.19	0.027

In order to further compare the dosimetric parameters of organ at risk for EDGE and CK, we calculated the radiobiological parameter EUD by the Eqs. (5)–(6) according the DVHs of spinal cord, bowel, stomach, duodenum, Liner, left kidney, right kidney and spleen, and the results are showed in Table 4. From the data of Table 4, the EUD values of spinal cord, duodenum, stomach, left and right kidneys are lower for CK plans, expect the liver having higher EUD value. And there are significantly statistic difference. But for bowel and spleen, both of two series plans have the similar value of dose-volume and no statistic difference.

**Table 4:** Comparison of the EUD for OARs between CK and EDGE plans.

Structure	α/β	*n*	EUD		
			CK + SD	Edge + SD	*p*
Spinal Cord	3	0.05	3.47 ± 1.27	6.37 ± 1.79	0.000
Duodenum	4	0.15	7.73 ± 2.29	9.12 ± 1.98	0.001
Bowel	4	0.15	10.34 ± 3.30	10.54 ± 3.75	0.722
Stomach	4	0.15	8.47 ± 3.59	12.41 ± 6.08	0.005
Liver	3	0.32	3.64 ± 1.62	2.86 ± 1.62	0.000
Left kidney	3	0.7	2.06 ± 0.73	3.06 ± 1.42	0.001
Right kidney	3	0.7	1.68 ± 0.49	2.50 ± 1.14	0.001
Spleen	3	0.5	1.67 ± 0.58	2.17 ± 1.72	0.159

### Dosimetric comparison with different ISDL

**Figure 6:**
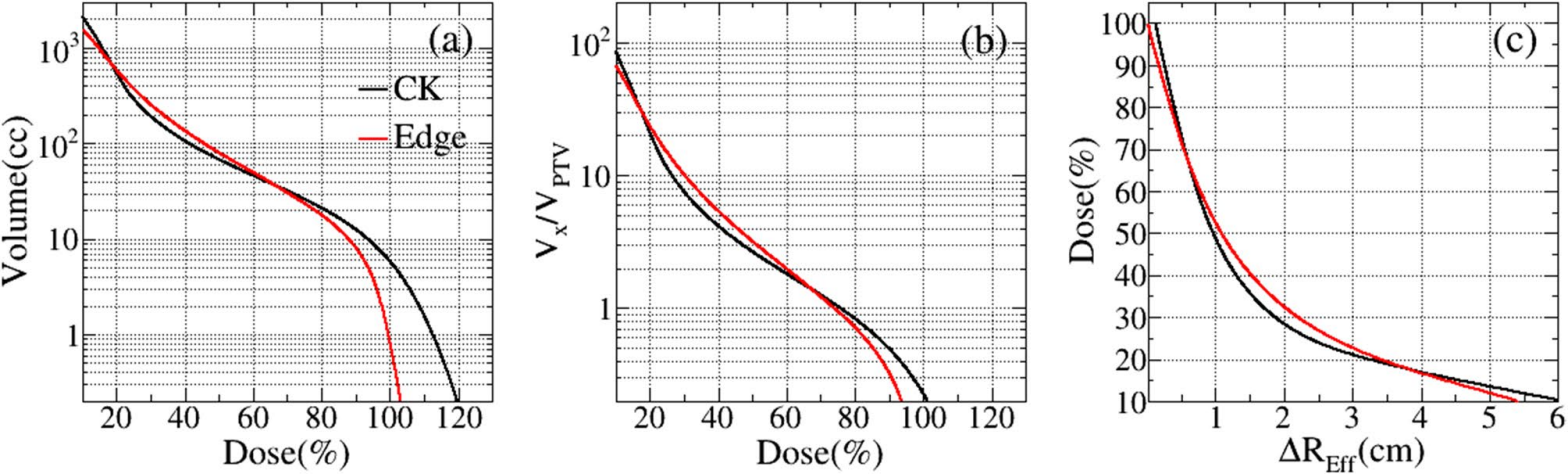
Comparison of normal tissue covered by different prescription isodose lines. (**a**) Absolute volumes (*V*_*x*_); (**b**) the volume ratios (*V*_*x/*_*V*_*PTV*_); (**c**) dose fall-off distance (*ΔR*_*Eff*_ ) for different isodose lines. The black line is for CK, and the red line is for EDGE.

The average volume of normal tissue covered by different prescription isodose lines are displayed in Fig. 6. In the Fig. 6a,b, the EDGE plans have the less volumes of normal tissue for the lower and higher prescription isodose region than CK plans, which provide the superiority to control the hot spot of tumor. These results are also in consistent with the Figs. 2 and 3. However, for the intermediate dose region with 20–70% of prescription isodose, it is obvious that the volume of normal tissue received radiation dose for CK plans are less than EDGE plans, as accordance with the Fig. 5 and Table 3. In the Fig. 6c, within the radius R of 0–1 cm, the CK plans showed the steeper dose fall-off gradient, as same the shown in Fig. 4. The average volumes, standard deviation (SD) and *p* values are listed in Table 5.

**Table 5:** Volume comparison of normal tissue covered by different isodose lines.

Isodose (%)	Volume (cc)			V_x_/V_PTV_			ΔR_Eff_ (cm)		
	CK ± SD	Edge ± SD	*p*	CK ± SD	Edge ± SD	*p*	CK ± SD	Edge ± SD	*p*
100	5.81 ± 3.06	0.83 ± 0.45	0.000	0.23 ± 0.06	0.04 ± 0.02	0.000	0.13 ± 0.04	0.02 ± 0.01	0.000
90	12.57 ± 5.32	8.11 ± 3.15	0.000	0.50 ± 0.08	0.33 ± 0.06	0.000	0.26 ± 0.05	0.18 ± 0.02	0.000
80	21.09 ± 8.33	17.83 ± 6.62	0.006	0.84 ± 0.10	0.72 ± 0.11	0.004	0.40 ± 0.06	0.35 ± 0.04	0.004
70	31.75 ± 12.25	30.39 ± 11.31	0.358	1.26 ± 0.13	1.22 ± 0.16	0.490	0.56 ± 0.08	0.54 ± 0.07	0.416
60	46.06 ± 17.62	49.55 ± 18.49	0.112	1.82 ± 0.17	1.98 ± 0.24	0.057	0.74 ± 0.11	0.78 ± 0.10	0.067
50	67.74 ± 25.89	79.79 ± 29.63	0.002	2.67 ± 0.22	3.19 ± 0.35	0.001	0.97 ± 0.15	1.09 ± 0.14	0.000
40	106.21 ± 40.78	135.81 ± 51.41	0.000	4.16 ± 0.30	5.40 ± 0.53	0.000	1.31 ± 0.20	1.53 ± 0.21	0.000
30	193.03 ± 74.99	255.94 ± 97.82	0.000	7.54 ± 0.65	10.16 ± 0.89	0.000	1.88 ± 0.30	2.21 ± 0.30	0.000
20	553.84 ± 264.31	590.73 ± 212.06	0.237	21.21 ± 4.91	23.58 ± 2.03	0.098	3.26 ± 0.67	3.42 ± 0.44	0.098
10	2158.22 ± 921.62	1584.76 ± 427.80	0.002	86.18 ± 16.20	67.59 ± 18.88	0.000	6.14 ± 0.91	5.43 ± 0.50	0.001

## Discussion

In this study, we made a plan quality comparison in terms of various dosimetric metrics for pancreatic cancer SBRT between Varian CyberKnife and EDGE systems. Both of the two techniques had the capability of producing clinically acceptable plans with adequate PTV coverage and OAR sparing. These results showed that EDGE plans offered more conformal and homogeneous dose distribution for PTV, while CK plans had slightly better dose coverage of PTV and the steeper dose fall-off gradient. For OARs, except D_5cc_ of bowel and the mean dose of bowel, stomach, liver, and kidneys are slightly lower for EDGE plans, the rest dose-volume metrics, as well as EUD were all lower for CK plans. When investigating the details of dose distribution outside PTV, it was obtained that the volumes covered by intermediate ISDL (ranging from 20 to 70%) were much lower for CK plans, while the EDGE plans indicated superior sparing for lower and higher dose region.

Our data indicated that the EDGE plans were more conformal and homogeneous compared to the CK plans. This may be related to the field arrangement and delivery techniques for different platforms. On the one hand, hundreds of non-coplanar field were used for CK plans while only two coplaner 360° arcs were applied for EDGE plans. This results in that the entire dose being deposited within the plane of the arcs for EDGE plans, while the radiation dose was concentrated in the center of the target area with much bigger degree of freedom for beam directions. At the same time, the hot spot in CK may be a litter larger than that of EDGE. On the other hand, the collimators of the two systems are also very different. CK plans only adopted 1–2 circular cones for beam shaping, but for EDGE system is equiped with high definition HD120 MLCs with spatial resolution of 2.5 mm^23^, which may made the better conformity and homogeneity of PTV for EDGE simultaneously, as shown in Figs. 1, 2 and 3.

For the two series of plans, the CK plans used the 6 MV FFF beams, while the EDGE chose the 10 MV FFF beams. When removing the flattening filter, it can offer increasing dose rate and make the beam profile more forward at the central axis. At the same time, there are other advantages for FFF beams, such as reduction of the scattered radiation and treatment head leakage^25^. With the 10 MV FFF modes, it could result in the relatively lower radiation dose exposure for OARs, as well as for the integrated body. However, in this study, the EDGE plans did not show any superiority for OAR sparing. Our previous study regarding to localized prostate cancer showed that EDGE plans not only provided more conformal and homogeneous dose distribution for PTV, but also steeper dose fall-off gradient and superior OAR sparing. The inconsistent results may partly due to the different shapes of PTV that affect the dose distributions. The shape of pancreatic cancer had a relatively regular shape, approximately ellipsoidal, so that both of the two series of plans were made to meet the dose constrains of PTV easily. In the Multiplan system, five ‘shells’ were applied to limit the dose outside PTV, which may lead to better normal tissue sparing. The delivery efficiency of beam is one of the most significant differences between the CK and EDGE systems. The average treatment time of per fraction was 2–3 min approximately for the EDGE, and 40–50 min approximately for the CK according to our clinical experience. On the one hand, the reduction of average delivery time can alleviate the discomfort of patients during radiotherapy. On the other hand, the effects of intra-fractional organs motion would be reduced by decreasing the treatment time for EDGE^36–38^.

Our results did show that a dose escalation of SBRT for pancreatic cancer in EDGE and CK systems both could reach the clinical criteria. But there are still some lmitations for this study. This study is a retrospective analysis and the SBRT plans for EDGE were not applied in clinical practice. Further studies were warranted to assess the clinical utility and radiobiological responses. Another limitations is that there is no consistent results for PTV margins and the organs motion[^38^, ^39^]. Whether patient specialized PTV margins could be obtain for different platforms, and how much the margins would affect the dose distribution for surrounding normal tissue will be the next tissue for our further study.

## Conclusion

A comparative quantitative assessment of the dosimetric and radiobiological indices of SBRT plans for 15 patients with pancreatic cancer between CK and EDGE systems.We confirm that radiotherapy systems with different characteristics should be investigated and utilized to help radiation oncologists choose a proper SBRT method for each individual patient to get better therapeutic effects. Although the CK system indicate better OAR sparing, the EDGE system can be regarded as an alternative option for SBRT of pancreatic cancer, especially for patients who cannot remain lying in bed for a long time.

## Data Availability

Not applicable.
